# Anti-Candida and Anti-Leishmanial Activities of Encapsulated *Cinnamomum verum* Essential Oil in Chitosan Nanoparticles

**DOI:** 10.3390/molecules28155681

**Published:** 2023-07-27

**Authors:** Rym Essid, Ameni Ayed, Kais Djebali, Houda Saad, Mondher Srasra, Yasmine Othmani, Nadia Fares, Selim Jallouli, Islem Abid, Monerah Rashed Alothman, Ferid Limam, Olfa Tabbene

**Affiliations:** 1 Laboratoire des Substances Bioactives, Centre de Biotechnologie de Borj-Cedria, BP 901, Hammam-Lif 2050, Tunisia; 2 Valorization of Useful Material Laboratory (LVMU), National Research Center in Material Sciences (CNRSM) Technopôle Borj Cedria, BP 73, Soliman 8027, Tunisia; 3 Centre National en Recherche en Sciences des Matériaux, “CNRSM” Technopole Borj-Cedria-Route Touristique Soliman, BP-273, Soliman 8027, Tunisia; 4 Department of Botany and Microbiology, College of Science, King Saud University, P.O. Box 2455, Riyadh 11451, Saudi Arabia

**Keywords:** nanoencapsulation, Cinnamomum verum essential oil, chitosan, ionic gelation, experimental design, anti-candida activity

## Abstract

Nanoencapsulation is widely considered as a highly effective strategy to enhance essential oils’ (EO) stability by protecting them from oxidative deterioration and evaporation. The present study aims to optimize and characterize an efficient technique for encapsulating *Cinnamomum (C.) verum* essential oil into chitosan nanoparticles using response surface methodology (RSM). Moreover, the optimized *C. verum* EO nanoparticle was investigated for its antibacterial (against Gram-positive and Gram-negative bacteria), antifungal (against *Candida albicans*), and antiparasitic activity (against *Leishmania* parasites). Five parameters were investigated using a Plackett–Burman and Box–Behnken statistical design: the chitosan molecular weight, TPP concentration, *C. verum* EO/chitosan ratio, mixing method, and the duration of the reaction. Encapsulation efficiency and anti-candida activity were considered as responses. The antibacterial, anticandidal, and anti-leishmanial activities were also assessed using a standard micro-broth dilution assay and the cytotoxicity assay was assessed against the macrophage cell line RAW 264.7. The optimized nanoparticles were characterized using Fourier transform infrared spectroscopy, Zeta potential, and scanning electron microscopy. The study results indicated that under optimal conditions, the nanoencapsulation of *C. verum* EO into chitosan nanoparticles resulted in an encapsulation efficiency of 92.58%, with a regular distribution, a nanoparticle size of 480 ± 14.55 nm, and a favorable Zeta potential of 35.64 ± 1.37 mV. The optimized *C. verum* EO/chitosan nanoparticles showed strong antifungal activity against *C. albicans* pathogens (CMI = 125 µg mL^−1^), notable antibacterial activity against both Gram-positive and Gram-negative bacteria (ranging from 125 to 250 µg mL^−1^), high leishmanicidal potential against the promastigotes form of *L. tropica* and *L. major* (IC50 = 10.47 and 15.09 µg mL^−1^, respectively), and a four-fold cytotoxicity reduction compared to non-encapsulated essential oil. These results suggest that *C. verum* EO-loaded chitosan nanoparticles could be a promising delivery system for the treatment of cutaneous *Candida albicans* infections.

## 1. Introduction

In the past few decades, the increasing number of various infectious diseases and the emergence of multidrug-resistant pathogens have prompted many researchers in the field of ethnopharmacology to discover new antimicrobial molecules. Some of the available synthetic drugs havevarious limitations, such as a lack of efficacy, toxicity, high costs, resistant strains, and drug interactions [1].

Infections caused by bacteria, fungi, or parasites are consistently on the rise [2] due to their ability to develop various resistance mechanisms against traditional medication.

*Candida albicans* is the most prevalent pathogen among fungal infections whichcauses serious invasive infections with high mortality and morbidity [3]. Under certain conditions, it can cause opportunistic infections, particularly in immunodeficient individuals. It serves as an important model organism for assessing the effectiveness of antifungal compounds. Furthermore, a parasitic disease such as leishmaniasis is a significant public health concern that has gained significant attention due to its widespread presence in endemic regions worldwide, particularly in tropical and subtropical areas [4]. The current treatments available against these pathogens are often expensive, toxic, and can lead to drug resistance [5].

In this regard, several plant extracts have attracted great interest as complementary and alternative chemical antimicrobials due to their cost-effectiveness and pharmacological activity. This is particularly true for essential oils (EO), which have received much interest in aromatherapy and are used in various fields in therapy, cosmetics, aromatics, fragrances, and spiritual uses [6]. They have been shown to possess a wide range of potent and safe compounds with a broad spectrum of antimicrobial properties [7].

One of the most used EO in aromatherapy is that of Cinnamon (*Cinnamomum verum*). It belongs to the laurel family (Lauraceae) and is known for its antibacterial, antioxidant, and antifungal activities [7,8]. These potencies are related to its main constituent, the phenylpropanoid cinnamaldehyde [3]. Nevertheless, the use of cinnamon EO is still limited by its high volatility, toxicity, low solubility in the aqueous phase, and instability under environmental or processing conditions [9].

Nanoparticles can be used as carriers for antimicrobial drugs, allowing targeted delivery to cells. This can improve the efficacy of the bioactive molecules and reduce toxicity and microbial resistance. The antimicrobial activity of nanoparticles stems from their small size, large surface area-to-volume ratio, and the ability to interact with microbial cells in multiple ways. Certain nanoparticles, such as silver nanoparticles, can interact with the bacterial cell membrane, leading to its disruption. This disruption can cause leakage of cellular components and ultimately cell death [10,11]. Other nanoparticles, such as titanium dioxide (TiO_2_) and zinc oxide (ZnO) nanoparticles possess photocatalytic properties [12]. When exposed to light, these nanoparticles can generate ROS, such as hydroxyl radicals and superoxide ions, which are highly reactive and can damage bacterial cells. Certain nanoparticles can inhibit the activity of specific enzymes in bacteria. For example, gold nanoparticles have been shown to inhibit the activity of bacterial enzymes involved in DNA replication and protein synthesis, thereby inhibiting bacterial growth [13]. Antibacterial activity has been extensively studied in various bacteria including Gram-positive and Gram-negative bacteria. These investigations have revealed the potential of nanoparticles and explored their effectiveness in treating a wide range of bacterial infections. Therefore, nanoparticles offer potential applications in combating several infections and contribute to the development of effective treatment options [10].

Nanoencapsulation of EO is an effective strategy to enhance the stability of EO and protect them from oxidative degradation and evaporation [6]. It is also a potent tool to improve the solubility, bioavailability, and controlled release of EO [14].

Chitosan, a natural polymer, is commonly used in pharmaceutical formulations because of its biodegradability, biocompatibility, and low cytotoxicity [10,11,12]. It also exhibitsantimicrobial properties against bacteria and fungi, making it an ideal delivery system [14]. Chitosan nanoparticles obtained through ionic gelation have been found to be effective in loading EO and improving their stability. The ionotropic gelation process is a simple, affordable, and solvent-free method to form stable EO-loaded chitosan nanoparticles, based on the electrostatic interaction between positively and negatively charged polymers [15].

In order to obtain an optimal EO nanoencapsulation in chitosan nanoparticles with improved encapsulation efficiency and anti-candida activity, the formulation parameters have to be carefully studied using response surface methodology (RSM) [16] and Box–Behnken design (BBD) [17]. To our knowledge, there have been no previous studies on optimizing *C. verum* EO nanoencapsulation in chitosan nanoparticles using PBD and BBD. While previous studies have shown the anti-infectious activity of *C. verum* EO [11], none have explored its antifungal and anti-leishmanial properties.

Therefore, the current study aims to explore the antifungal and antiparasitic activity of *C. verum* EO loaded into chitosan nanoparticles using Placket–Burman and Box–Behnken response surface methodology. The characterization of the obtained optimal nanosystem and the evaluation of the cytotoxic activity were also studied.

## 2. Results and Discussion

### 2.1. C. verum EO Yield and Chemical Composition

The yield of extracted cinnamon oil from dried bark plant material was 0.80 ± 2%. The composition of *C. verum* EO was investigated by gas chromatography-mass spectrometry (GC-MS). Among the phytochemicals, the phenylpropanoid cinnamaldehyde was the most abundant with a value of 82.09%. The monoterpene oxide 1,8-cineole and the sesquiterpene α-copaene were found at lower proportions (3.1% and 3.06%, respectively). In general, the relative chemical composition varies remarkably with geographical location, climate, harvesting time, and extraction technique [18]. This result is in complete agreement with previous reports [3]. Ainane et al. [19] showed that cinnamaldehyde is the major compound of *C. verum* (89.31%). However, Valizadeh et al. [20] reported a lower proportion of cinnamaldehyde (69.15% and 57.83%, respectively).

### 2.2. Determination of Significant Factors by Plackett–Burman Design

The nanoencapsulation of *C. verum* EO in chitosan nanoparticles was first optimized by Plackett–Burman design in order to identify significant factors involved in the encapsulation process. Briefly, 14 experiences and 2 responses (encapsulation efficiency and anti-candida activity) were evaluated (Table S1). The experiences from 9 to 14 allow for the calculation of the experimental variance to determine the significant factors.

The encapsulation efficiency percentage of cinnamon EO was significantly (*p* < 0.05) influenced by the independent variables: chitosan MW, TPP concentration, EO /chitosan ratio, and mixing method. However, the variable time (h) did not show any significant effect (Table S2). Moreover, all factors were highly significant (*p* < 0.01%) for the anti-candida activity response (*Y2*). The method *X1* had a positive effect on *Y1* but a negative effect on *Y2* responses. Therefore, it was set at a high level (+1). However, factor *X2*(chitosan MW) had a negative effect on both *Y1* and *Y2* responses and was therefore fixed at its low level (−1).

According to the Box–Behnken response surface method (RSM) and the analysis of variance (ANOVA), the independent significant factors: TPP concentration, *C. verum* EO/chitosan ratio, and the reaction time were evaluated at three coded levels (Table S3): high (+1), medium (0), and low (−1). In total, 20 experiments were conducted.

Table S4 summarizes the significance of the factors and their interactions. Indeed, only coefficients a4, a5, a55, a34, and a35 have a significant effect on the encapsulation efficiency (*Y1*) (*p* < 5%), while all the factors and their interactions are highly statistically significant (*p* < 1%) for “anti-candida activity” response (*Y2*).

The interaction effect of significant factors on the responses anti-candida activity and EE % is shown in Figure S1. The significant factors were [TPP] (*X3*), the ratio EO /chitosan (*X4*), and the reaction time (*X5*). The interaction between TPP and the EO /chitosan ratio showed that optimal anti-candida activity (125 µg mL^−1^) was observed at high levels of both factors (1 mg mL^−1^ and 2/1, respectively). In addition, optimal anti-candida activity was observed at a high reaction time and a high ratio of EO/chitosan (2 h and 2/1, respectively). Moreover, an optimal encapsulation efficiency response was observed at a high level of TPP, of the ratio of EO/chitosan, and the reaction time.

To validate the optimization as predicted by the desirability analysis, a checkpoint analysis was performed with the optimal range (Figure S2). The optimized conditions showed an encapsulation efficiency of 93 ± 3% and anti-*Candida* activity of about 120 ± 10 µg mL^−1^. These results were very close to the predicted responses (Table S5), therefore demonstrating the accuracy of the predicted optimum conditions.

Previous studies outlined the importance of the statistical analysis models PBD and BBD to enhance the encapsulation efficiency of essential-oil–loaded nanoparticles [15,16]. Almeida et al. [15] proved that the EE% increased by optimizing several factors such as the *Cymbopogon citrates* EO/PLGA–nanoparticles ratio, sonication time, and ultrasound power using a BBD design. Similarly, the increasing amount of jasmine oil, using BBD design, improved the EE% [16]. However, the results of Matshetshe [21] noted a lower EE of cinnamon EO in CN nanoparticles (ranging from 10.12 to 20.04%). However, it decreases at higher EO levels. Furthermore, the microencapsulationof eucalyptus EO was also optimized by RSM and showed a significant effect of the temperature and incubation time on EE% [22].

The EE% was shown to be dependent on the amount of EO, TPP concentration, and encapsulation time. In fact, the encapsulation efficiency of cinnamon EO in CN-NPs decreased from 20.04 to 10.12% at a higher ratio of chitosan/EO [21].

The use of a negatively charged phosphate group of TPP is necessary to form cross-links with positively charged amino groups of chitosan by ionic interaction. In this sense, the TPP proportion should be optimized [23]. According to Yongmei and Yumin [24], the formation of nanoparticles occurs only at a certain concentration of CN and TPP. It was proven by [6] that the highest encapsulation of cardamom EO in chitosan was obtained by using 0.1% TPP.

### 2.3. Biological Activity Determination

1. Anti-candida Activity

Based on the RSM results, the optimal preparation conditions were defined as follows: TPP concentration: 1 mg mL^−1^, ratio EO/chitosan: 2/1, and reaction time: 2 h. *C. verum* EO and *C. verum* EO/CN-NPs showed effective activity against *C. albicans* strains at MIC values of 62.5 µg mL^−1^ and 125 µg mL^−1^, respectively (Table 1).

Previous studies reported that *C. verum* demonstrated anti-candida activity, with a minimum inhibitory concentration (MIC) value of 1 mg mL^−1^ against *C. albicans* ATCC 5314 and *C. tropicalis* ATCC 750 [25]. On the other hand, *C. zeylanicum* exhibited a MIC value of 312.5 µg mL^−1^ against *C. albicans* ATCC 40277 [26]. The variation in these results can be attributed to differences in the composition of EO and the specific Candida species tested.

Regarding encapsulation, several EO such as oregano, *Carum copticum*, clove, cinnamon, *Eucalyptus staigeriana,* and those containing limonene have been successfully encapsulated using the ionic gelation technique [27,28,29,30,31]. Since Chitosan is the most suitable encapsulation polymer because of its natural abundance, biodegradability, and surface functional groups with free NH_2_ groups, it has been largely used to encapsulate EO [12]. Moreover, it has been shown that chitosan nanoencapsulation is a good tool to maintain the antioxidant and antimicrobial properties of several EO [32]. Encapsulated jasmineoil (JO) also exhibited excellent cyto-compatibility against normal cells and demonstrated better antioxidant and anticancer properties than free JO [16,33].

Nanoencapsulation of *C. verum* into chitosan has already been used as a potential preservative against mycotoxins for conservation [34] and in the diet of broiler chickens to enhance their intestinal bacteria population and, consequently, their immune responses [35]. However, no reports have investigated the anti-candida activity of *C. verum* EO. Recent anti-candida drugs, suchas1-amino-5-isocyanonaphthalene (ICAN), its dimethylated derivative (DIMICAN), and 1,5-disocyanonaphtalene (DIN) have show good anti-candida activity, with MIC values between 0.6 and 5 μg mL^−1^ [36].

2. Antibacterial Activity

The essential oil (EO) of *C. verum* demonstrates notable antibacterial activity, ranging from 125 to 250 µg mL^−1^, against both Gram-positive and Gram-negative bacteria. According to the findings reported in Table 2, the use of *C. verum* EO/CN-NPs resulted in enhanced antibacterial activity. Formed nanoparticles exhibit stronger antibacterial effects against Gram-positive bacteria compared to Gram-negative bacteria. In fact, they demonstrate an inhibition zone (IZ) of 16 mm and MIC values of 62.5 µg mL^−1^ against Gram-positive bacteria (*Staphylococcus aureus* ATCC 6538 and *Listeria monocytogenes* ATCC 19115, respectively). In addition, they showed an IZ of 14 mm and MIC values of 125 µg mL^−1^ against Gram-negative bacteria (*Escherichia coli* ATCC 25922 and *Salmonella enteritidis* DMB 560) (Figure S3).

Several studies have investigated the antimicrobial properties of cinnamon. Barbarossa et al. [37] reported lower antibacterial activity against *Staphylococcus aureus* ATCC 25923 and *Escherichia coli* ATCC 25922 (CMI of 1.22 and 4.88 mg mL^−1^, respectively). Gupta et al. [38], reported that cinnamon EO showed a MIC value ranging from 1.25% to 5% *v*/*v*. It exhibited the strongest effect on *Bacillus cereus* (bacteria). Furthermore, a recent study assessed the anti-biofilm effects of cinnamon EO and liposome-encapsulated cinnamon EO on methicillin-resistant *Staphylococcus aureus* (MRSA). The analysis, using scanning electron microscopy and laser scanning confocal microscopy, revealed that cinnamon EO displayed effective antibacterial and prominent anti-biofilm activities against MRSA. The presence of liposomes further improved the stability and duration of action of cinnamon EO [39].

The main active component of cinnamon, cinnamaldehyde, has been found to exert antimicrobial effects on microorganisms by inhibiting cell wall biosynthesis, membrane function, and specific enzyme activities [40]. Additionally, chitosan demonstrates antimicrobial properties due to its high cationic charge, enabling interaction with the negatively charged bacterial cell membrane. This interaction results in the depolarization of bacterial cell membranes, increased permeability, and ultimately, cell lysis [41].

3. Anti-leishmanial activity

*C. verum* EO displayed high anti-promastigote activity against *L. tropica* and *L. major* (IC_50_ = 14.11 and 17.32 µg mL^−1^, respectively), with an inhibition growth of more than 90% (Table 3). An improvement in this activity was recorded with *C. verum*/CN-NPs which exhibited high anti-promastigote activity toward *L. tropica* and *L. major* (IC_50_ = 10.47 and 15.09 µg mL^−1^, respectively).

Interestingly, no cytotoxicity was recorded for the encapsulated *C. verum* EO toward macrophage cell line RAW 264.7 compared to free *C. verum* EO (SI = 95.5 and 66.26 against *L. tropica* and *L. major,* respectively) (Table 3). Thus, our results have outlined the effectiveness of nanoparticles as leishmanicidal agents and their harmlessness to macrophages (SI > 10).

Other studies on *Cinnamomum zeylanicum* EO have shown an IC50 of 16.53 and 7.56 μg mL^−1^ against *L. major* and *L. tropica* promastigotes, respectively [42].

Many new studies have emerged with nanoparticles serving as promising therapeutic agents for anti-leishmanial disease treatment. Liposomal amphotericin B (AMB) is one of the successful nano-based drugs with high efficacy and negligible toxicity.However, there are no reports of nanoencapsulation for *Cinnamomum* spp. EO nor any information about its anti-leishmanial activity.

Recently, numerous studies highlighted the potential of nanoparticles as a promising therapeutic approach for the treatment of anti-leishmanial diseases. Among these nano-based drugs, liposomal Amphotericin B has demonstrated remarkable efficacy and minimal toxicity [43]. However, there is a lack of research on nanoencapsulation techniques for *Cinnamomum* spp. EO, as well as a dearth of information regarding its anti-leishmanial activity.

4. Cytotoxicity against Raw 264.7

Our results are in accordance with other studies showing that EO-CN-NPs reduced the cytotoxicity of EO compared with free EO [6,15,44]. Furthermore, chitosan nanoparticles are considered as a non-toxic matrix as they did not show any toxicity effect on cell lines, and 100% cell viability was recorded on HCEC and HepG2 cell lines [45]. Likewise, no hemolytic activity was recorded for CN-NPs [18,46].

Even though 64.1 % of the EO was released after 72 h of incubation, no toxicity was recorded. These findings suggest that chitosan-loaded EO may have potential applications in protecting cells from damage and reducing toxicity, making them a promising area for further research and development [47]. In fact, it was demonstrated that the positive charges of chitosan amino groups (NH3^+^) interfere with the negative charges of the cell membrane, inducing their agglomeration and preventing their damage and hemolysis [48]. Moreover, nanoencapsulation is based on the protection of cells from the formation of degraded compounds that can have toxic derivatives [49]. Moreover, several other factors may affect cytotoxicity including the chitosan concentration, its molecular weight, and its degree of deacetylation (DD) [50]. Polymers with high cationic charge densities have higher cytotoxicity than those with low charge densities. Interestingly, the present study showed a low cationic charge density (35 mV), leading to low cytotoxic activity.

In addition, the structure of nanoparticles affects the cytotoxicity. The size, concentration, and Zeta potential of particles play crucial roles in determining the cytotoxicity of nanoparticles [51]. Similarly, Hu et al. [52] showed that nanoparticles can selectively transport essential oils (EO) to target cells without affecting healthy cells and gradually release them by changing the pH in the target environment. This selective targeting and controlled release of EO can increase their therapeutic potential and reduce their toxicity. Similarly, Qi et al. [53] reported that chitosan nanoparticles exhibit selective inhibitory activity against various tumor cell lines while showing low toxicity against normal human cells. These findings highlight the potential of chitosan nanoparticles as a delivery system for essential oils, with the ability to selectively target cancerous cells while minimizing their impact on healthy cells.

Overall, these studies demonstrate the potential of nanoparticles, particularly chitosan nanoparticles, as a delivery system for essential oils and their ability to selectively target cells and reduce toxicity, making them a promising area for further research and development.

### 2.4. Invitro Release of C. verum EO

**Figure 1:**
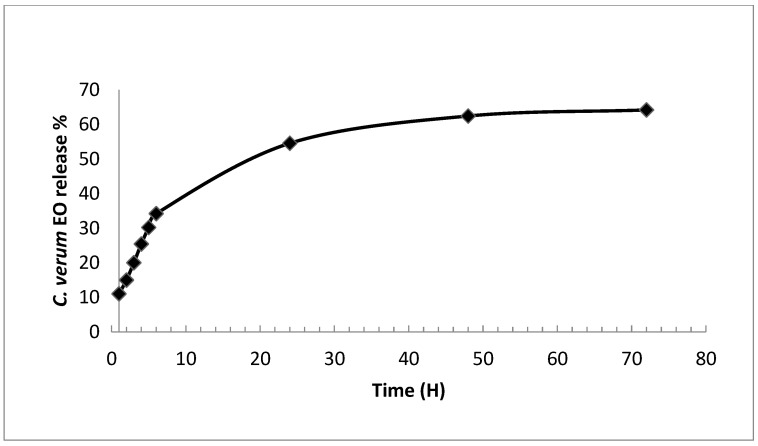
In-vitro release profile of *C. verum* EO from CN NPs.

The In-vitro release kinetics of *C. verum* EO from CN-NPs was determined in 20% ethanol and incubated at 30 °C. As shown in Figure 1, the release of *C. verum* EO from CN-NPs started at a fast rate and reached 54.55% release within 24 h. Subsequently, a slow cumulative release rate was recorded, reaching 62.4 ± 2.3% and 64.1 ± 2.6% release at 48 h and 72 h incubation, respectively. It could be suggested that the initial burst release might be attributed to the EO molecules adsorbed on the surface of CN-NP and oil entrapment near the surface [24]. The second stage was characterized by a slow release rate which might be related to the diffusion and the dispersion of the EO into the polymer matrix. This controlled release helps maintain its biological activity. Similar behavior for the biphasic release profile was observed for oregano, cumin, and *Anethum graveolens* EO from chitosan nanoparticles [46]. Polymeric nanoparticles based on chitosan are considered as an excellent drug carrier for the controlled release of the drug. Noteworthy, drug release from a CN-based nanoparticle system depends on the morphology, size, and density of the system. It depends also on the physicochemical properties of the drug as well as the presence of adjuvants. In fact, the in-vitro release of the drug from CN-NPs depends on pH, polarity, and the presence of enzymes in the dissolution system [54].

### 2.5. Particle Size and Zeta Potential Determination

The Zeta potential of both chitosan/TPP (CN-NPs) and the optimum of *C. verum* EO/CN-NPs was determined (Table 4). Both of them displayed positive Zeta potential values related to the protonation of the NH_2_ group under acidic conditions [55]. Furthermore, a decrease in Zeta potential charge (from 42.57 ± 0.87 mV for CN-NPs to 35.64 ± 1.37 mV for *C. verum* EO/CN-NPs) was observed. This decrease may be explained by the adsorption of the EO on the surface of CN-NPs. The characterization of all formed nanoparticles was added to S1 (supplementary data).

These results are in good agreement with those of Sotelo-Boyás and collaborators [41], who suggested that the decrease of the Zeta potential was attributed to the decrease of free NH_3_^+^ groups of chitosan after interaction with lime EO. A Zeta potential value greater than 30 mV makes nanoparticles repel each other and ensures the physical colloidal stability of the suspension [6]. Moreover, the positive charge of both empty and loaded CN-NPs indicated prompt interaction with bacterial pathogens [55].

The mean size of CN-NPs and EO/CN-NPs was also measured. The results showed that the particle size of CN-NPs was 210 ±10.32 nm. The *C. verum* EO/CN-NPs size increased, reaching 480 ±14.55 nm. These results could confirm the incorporation of EO into CN-NPs. Similar results were reported by Ghahfarokhi et al. [12], showing that the addition of Cinnamon EO resulted in an increase in the CN-NPs’ diameter. Additionally, the mean particle size of the CN-NPs increased with the amount of encapsulated peppermint oil [56]. It has been reported that CN-NPs’ size depends on the nanoencapsulation method [57]. In fact, the use of a TPP concentration from 0.05 to 1 mg mL^−1^ allows for the formation of very small CN-NPs [41], while increasing the TPP concentration above 1 mg mL^−1^ resulted in the formation of aggregates [58].

**Figure 2:**
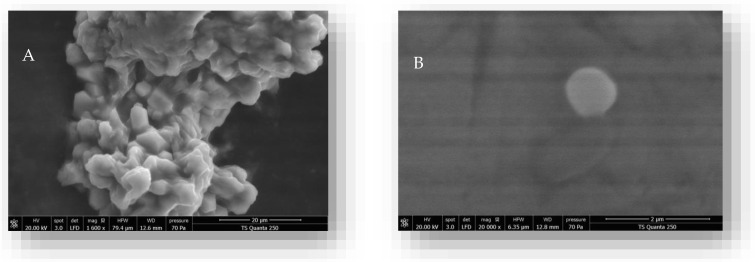
Scanning electron microscopy of CN-NPs (**A**) and *C. verum* EO/CN-NPs (**B**).

Furthermore, the scanning electron microscopy image of *C. verum* EO/CN-NPs observed in Figure 2 shows spherical-shaped particles with a well-defined structure and regular distribution with the absence of cracks.

### 2.6. Fourier Transform Infrared (FTIR) Spectroscopy

**Figure 3:**
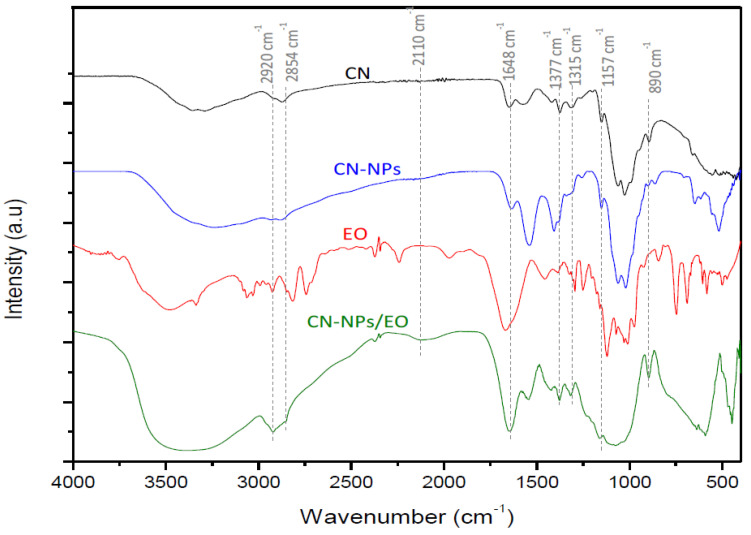
FTIR spectrum of unloaded chitosan (CN), chitosan nanoparticles (CN-NPs), *C. verum* essential oil (EO), and encapsulated EO into chitosan nanoparticles (*C. verum* EO/CN-NPs).

FTIR analysis was used to confirm the formation of chitosan nanoparticles and the encapsulation of *C. verum* EO into CN-NPs (Figure 3). The infrared spectrum of chitosan nanoparticles showed characteristic peaks at 1648 cm^–1^ corresponding to the amide I band, 1530 cm^–1^ for the amide II band (-NH_2_ bending), and 1066 cm^–1^ (P=O), indicating the formation of an ionic complex via the electrostatic interactions between the NH3 ^+^ groups of CN and the phosphate groups of TPP within the CN-NPs.

Moreover, the infrared spectra of the *C. verum* EO/CN-NPs showed absorption bands corresponding to the =C-H bond at 2920 cm^–1^, the C-H bond of the carbonyl group at 2845 cm^–1^, the carbonyl group C=O between 1500 cm^–1^ and 1648 cm^–1^, and the C=C bond between 1315 cm^–1^ and 1377 cm^–1^. The band at 890 cm^–1^ was assigned to the C-H bending of the aromatic ring. These results suggested the successful loading of cinnamon EO in the CN-NPs.

The mechanism of this interaction can be explained by the intermolecular hydrogen bonding and electrostatic interaction between the positive charge of the amino groups (NH_2_) in the CN and the negative charge of the carboxyl (COOH) groups in the *C. verum* EO on the NPs’ surface. These interactions were identified by the FTIR spectrum and by the formation of new bands corresponding to the C-H stretching at 2920 cm^–1^ and 2845 cm^–1^, the C=O stretching between 1500 cm^–1^ and 1648 cm^–1^, and the C=C band between 1315 cm^–1^ and 1377 cm^–1^. Similar results were reported by Hu and coll [39].

## 3. Conclusions

In summary, the current investigation has demonstrated that encapsulating *C. verum* EO into chitosan nanoparticles is an effective method to enhance the EO’s stability, extend its efficiency for a long period, and reduce its cytotoxicity. The use of Plackett-Burman and Box-Behnken statistical designs in formulating the *C. verum* EO/CN-NPs has been successfully established, showing high EO encapsulation efficiency and a regular nanoparticles distribution. In addition, the obtained nanoparticles have been proven to be advantageous in controlling and reducing bacteria (against both Gram-positive and Gram-negative bacteria), fungal (against *C. albicans*), and parasitic infections (specifically against the *Leishmania* parasite). This strategy will exhibit promise in the pharmaceutical and food industries. Further studies are needed to assess the in vivo potency of this bioactive nanocarrier and evaluate its mechanism of action.

## 4. Material and Methods

### 4.1. Materials

The chitosan with different molecular weights, low (LMW), medium (MMW), and high (HMW), pentasodium tripolyphosphate (TPP), and acetic acid were purchased from Sigma-Aldrich (St. Louis, MI, USA). The *Cinnamomum (C.) verum* EO was purchased from a local market in Tunisia, in the form of dried bark strips, as previously described [3]. Amphotericin B (AMB) was used as a conventional anti-candida and anti-leishmanial treatment.

The bacterial strains including the Gram-negative bacteria (*Escherichia coli* ATCC 25922 and the clinical strain *Salmonella enteritidis* DMB 560) and Gram-positive bacteria (*Staphylococcus aureus* ATCC 6810 and *Listeria monocytogenes* ATCC 19115), the yeast strain *Candida (C.) albicans* ATCC 10231, the Leishmanial strains (including *L. tropica* and *L. major*), and the murine macrophage Raw 264.7 cell line were obtained from the microorganism collection of the Bioactive Substances Laboratory, CBBC, Tunisia.

### 4.2. Essential Oil Extraction

Cinnamon EO was extracted by hydro-distillation in a Clevenger-type apparatus for 4 h as recommended by the European Pharmacopoeia [59]. Approximately 150 g of the dried bark was hydro-distilled. Due to the different polarity, the essential oil was separated from the hydrolates by simple decantation. The cinnamon EO obtained was concentrated, dried with anhydrous sodium sulfate, and stored in sealed amber glass vials at −20 °C until use. The percentage yield of cinnamon EO was calculated using the following Formula (1):

**EO yield = (EO weight/weight of Cinnamon powder) × 100** (1)

### 4.3. GC-MS Analysis of C. verum EO

The *C. verum* EO was analyzed by gas chromatography-mass spectrometry (GC-MS) using a gas chromatograph HP 7890 (II) and an HP 5975 mass spectrometer (Agilent Technologies, Palo Alto, CA, USA). Components separation was performed using capillary column HP-5 MS (30 m × 0.25 mm, 0.25 μm film thickness; Agilent Technologies, Hewlett-Packard, CA, USA). Helium was used as a carrier gas with a flow rate of 1.2 mL min^−1^, a split ratio of 60:1, and a scan time and mass range of 1 s and 40–300 *m*/*z*, respectively. The mass spectrometer was performed with electron ionization at 70 eV. The ion source was programmed at a temperature of 40 to 280 °C at a rate of 5 °C min^−1^. Compound identification was carried out through a comparison of the recorded mass spectra with those stored in the mass spectra library Wiley 09 NIST 2011 provided by the GC-MS data system.

### 4.4. Preparation of C. verum-EO/CN-NPs

*C. verum* EO incorporated into chitosan nanoparticles (CN-NPs) was prepared using the ionic gelation method, as previously described [6]. Briefly, chitosan was dissolved in 0.1% aqueous acetic solution (*v*/*v*) under magnetic stirring for 24 h. CN-NPs were prepared by adding a TPP cross-linker. Synthesis of *C. verum* EO/CN-NPs was performed, as previously described [60]. *C. verum* was added to the chitosan solution under moderate magnetic stirring at room temperature. The nanoparticles were collected spontaneously after centrifugation at 16,000 rpm for 30 min at 4 °C and then dissolved in distilled water and treated with a vortex mixer to obtain an homogenized dispersion.

### 4.5. Experimental Design

To optimize the nanoencapsulation of *C. verum* EO into chitosan nanoparticles, five variables were examined using the Plackett–Burman design (PBD). Then, the Box–Behnken design (BBD) was used to determine the effect of the selected significant factors on the responses: encapsulation efficiency (EE%) and anti-candida activity.

#### 4.5.1. Plackett–Burman Screening Design Methodology

Five independent factors were evaluated using PBD: the chitosan MW (*X1*), the TPP concentration (*X2*), the EO/chitosan ratio (*X3*), the mixing method (*X4*), and the duration of the reaction (*X5*). For each independent variable, three levels were evaluated: low (−1), medium (0), and high (+1). The responses were the EE% (*Y1*) and the anti-candida activity (*Y2*) (Table S6). The PBD was used to correlate dependent and independent variables using the following linear model (2):

(2) $$Y=A_{0\;}+\sum A_{i}X_{i}\;\;\;(i=1,\ldots \ldots ..,K)$$

where *Y* is the response, *A_0_* is the average of all responses, *A*_1_ − *A_K_* are the coefficients of the input parameters, and *X_i_* is the level of the independent variable. The significance of each parameter was determined using Student’s *t*-test.

#### 4.5.2. Box–Behnken Design (BBD) Response Surface Methodology (RSM)

The purpose of the response surface methodology (RSM) was to predict and explore optimal conditions for the nanoencapsulation of cinnamon EO into chitosan with optimal anti-candida activity and high encapsulation efficiency. In this design, three levels were required (−1, 0, and +1) to fit the second-order model. Data processing of the Box–Behnken design was performed using Nemrod W experimental design software. The significant factors of chitosan (%), TPP (%), and EO concentration (mg) were taken as variables while the non-significant factors were fixed at their economic level. Anti-candida activity and encapsulation efficiency were taken as responses.

The interaction between the independent variables and the responses was assessed using the second-order mathematical polynomial regression model (3), which isexpressed as follows:

(3) $$Y_{cal}=a_{0}+\sum_{i}a_{i}X_{i}+\sum_{ij,i\neq j}a_{ij}X_{i}X_{j}+\sum_{ii}a_{ii}X_{i}^{2}$$

where *Y_cal_*: the predicted response; *a_0_*: the intercept; *a_i_*: the linear coefficients; *a_ii_*: the square coefficients; *a_ij_*: the interaction coefficients; and *X_i_*, *X_j_*: the independent variables.

#### 4.5.3. Multi-Response Optimization Using Desirability

The desirabilityfunction (D) is an effective approach to the numerical optimization of the process with multiple responses. It is estimated as the geometric mean of the individual desirability functions and is given by Equation (4):

(4) $$D=\sqrt[{m}]{\prod_{i}^{m}d_{i}}$$

***m*** is the number of responses chosen (two responses in our case: encapsulation efficiency (Y1) and anti-candida activity (Y2)).

The desirable value ranges from 0 (undesirable value) to 1(the most desirable value). For each response, the desiredobjective is selected from the menu of the NemrodW software (LPRAI version 2000).

### 4.6. Anti-Candida Assay

The antifungal activity of *C. verum* EO and *C. verum* EO /CN-NPs was investigated against *C. albicans*ATCC 10231 using the microdilution method in 96-well plates [61]. Briefly, serial two-fold dilutions of samples (from 62.5 to 2000 µg mL^−1^) were added to 10^5^ cells mL^−1^ of yeast suspension and incubated for 24 h. The minimum inhibitory concentrations (MICs), defined as the lowest concentration of the active ingredient that inhibited *Candida albicans* growth, were determined by the addition of MTT solution (10 mg mL^−1^).

### 4.7. Antibacterial Activity

#### Well Diffusion Method

The antibacterial activity of *C. verum* EO nanoparticles was assessed using the agar well diffusion method, as previously described [62]. In brief, bacterial suspensions were prepared in PBS to achieve 0.5 McFarland standards (approximately 1–2 × 10^8^ CFUmL^−1^). These suspensions were then spread evenly on agar plates using sterile swabs. Next, the samples were placed into agar wells in Mueller–Hinton (MH) broth and incubated at 37 °C for 24 h. The presence of antibacterial activity was indicated by measuring the diameter of the inhibition zones (IZs) surrounding the wells.

5. Minimum inhibitory concentration determination (MIC):

The micro-dilution broth assay was used using 96-micro-well plates to determine the MIC value of *C. verum* EO nanoparticles, as previously described [63]. Briefly, two-fold serial dilutions of the samples ranging from 62.5 to 2000 µg mL^−1^ were added to bacterial suspensions (2 × 10^4^ CFUmL^−1^). The plates were then incubated at 37 °C for 24 h. The MIC, defined as the lowest concentration of the active ingredient that inhibited microbial growth, was determined by adding an MTT solution (10 mg mL^−1^).

### 4.8. Leishmaniacidal Activity

The in vitroleishmaniacidal potential was assessed against two Leishmania species responsible for cutaneous leishmaniasis: *L. tropica*and *L. major*. In brief, stationary promastigotes forms (2 × 10^5^cells) were cultured in 96-well culture plates for 72 h at 26 °C in the presence of different concentrations of *C. verum* EO and *C. verum*/CN-NPs. Cell viability was evaluated using the colorimetric assay method with 3-(4.5-dimethylthiazol-2-yl)-2.5-diphenyl tetrazolium bromide (MTT, Sigma-Aldrich). The optical density (OD) values were measured using a microplate spectrophotometer (Biotech-Synergy) at 570 nm [64]. The inhibitory concentration of 50% (IC_50_), corresponding to the concentration that reduces 50% of the parasite viability, was determined by applying a sigmoidal regression of a dose–response curve. AMB (Sigma-Aldrich) was used as a drug control. All assays were performed in triplicate.

### 4.9. Cytotoxicity Assay and Selectivity Index

The cytotoxicity assay of free and encapsulated *C. verum* EO in CN-NPs was assessed invitro on the murine macrophage Raw 264.7 cell line. Macrophage cells at 2 × 10^5^ cells/well were added into a 96-well tissue culture plate and allowed to adhere overnight in a growth medium consisting of RPMI-1640 medium (Sigma-Aldrich R 1383). Two-fold serial dilutions of samples (62.5 to 2000 μg mL^−1^) were added into the cells and incubated at 37 °C for 72 h. Cell viability was determined using the MTT (3-(4,5-Dimethylthiazol-2-yl)-2,5-diphenyltetrazolium bromide) assay, as described above [19]. Briefly, 100 μL of MTT (1 mg mL^−1^) wasadded to each well and incubated for 4 h at 37 °C. Subsequently, the supernatant wasremoved, and the MTT crystals were dissolved in DMSO (dimethylsulfoxyde). The amount of formed formazan wasdetermined spectrophotometrically at 570 nm.

The cytotoxicity expressed as CC50 corresponds to the treatment concentration causing 50% of cell death [3]. The selectivity index (SI) was also calculated as the ratio of the toxic concentration (CC50), and the selectivity index (SI) was determined as the ratio of the CC_50_ macrophage/IC_50_ parasite [64].

### 4.10. Determination of the Encapsulation Efficiency (EE%)

The encapsulation efficiency of *C. verum* EO/CN-NPs was evaluated as previously proposed [65]. In brief, the residual EO in the aqueous phase (free EO) was quantified using UV-Vis spectroscopy at 280 nm (Synergy, Bioteck) and deducted from the total amount of EO used in the encapsulation process. The encapsulation efficiency was calculated using Equation (5) as follows:

**Encapsulation efficiency = [(Total amount of EO − free EO)/Total amount of EO] × 100** (5)

The concentration of EO was calculated using a calibration curve generated from cinnamaldehyde, the main compound of *C. verum* EO, within a specified concentration range (r^2^ = 0.999). All analyses were carried out in triplicate.

### 4.11. In-Vitro Release Studies of C. verum EO

The *in-vitro*release study of *C. verum* EO from CN-NPs was conducted as previously described [66]. Briefly, 20 mg mL^−1^ of *C. verum* EO/CN-NPs wasdispersed in 20% ethanol and incubated at 30 °C. At specific time intervals (0, 1, 2, 3, 4, 6, 24, 48, and 72 h), the samples were centrifuged for 30 min at 16,000 rpm. The supernatant was then collected and replaced with an equivalent amount of 20% ethanol. The amount of EO released was measured spectrophotometrically at 280 nm using a calibration curve as mentioned above. The cumulative percentage of EO released was defined as the ratio of the cumulative amount of EO at each sampling time and the total amount of EO.

### 4.12. Characterization of C. verum EO/CN-NPs

Optimal *C. verum* EO/CN-NPs was characterized by Fourier transform infrared spectroscopy (FTIR) and dynamic light scattering.

#### 4.12.1. Fourier TransformInfrared Spectroscopy (FTIR)

Fouriertransform infrared absorption was recorded using an FTIR spectrometer (Frontier Perkin Elmer). This technique is based on the measurement of the infrared (IR) absorption and emission spectra of most materials to reveal newly formed functional groups and chemical bonds between the EO and CN-NPs [58]. The spectra of cross-linked chitosan nanoparticles without EO were compared with EO-loaded cross-linked chitosan nanoparticles. The spectra were also scanned over the wave number range from 400 to 4000 cm^−1^.

#### 4.12.2. Zeta Potential and Particle Size Measurements

The particle size, Zeta potential, and size distribution using the polydispersity index (PDI) of formed nanoparticles were determined by dynamic light scattering (DLS) using a Malvern Zeta sizer Nano ZS instrument and Zeta-sizer software (Malvern Instruments, UK). In addition, the morphology and the shape of the formed nanoparticles were studied using a scanning electron microscope (SEM, JSM-5400 JEOL) at 10 kV [55].

## Figures and Tables

**Table 1:** Anti-candida activity and cytotoxicity of CN-NPs and *C. verum* EO/CN-NPs opt.

	*C. albicans* MIC ± SD (µg mL^−1^)
CN-NPs	>2000
*C. verum* EO	62.5 ± 1.45
*C. verum* EO/CN-NPs opt	125 ± 0.66
AMB	2 ± 0.03

CN-NPs: chitosan nanoparticles. *C. verum* EO: *Cinnamon verum* essential oil. AMB: amphotericin B. The values presented are the mean of three replicates (*n* = 3) ± standard deviation (SD). AMB: amphotericin B.

**Table 2:** Antibacterial activity of *C. verum*EO and *C. verum* EO/CN-NPs.

	IZ (mm) /MIC (µg mL^−1^)						
Gram-Positive Bacteria	*C. verum* E0		CN-NPs		*C. verum* EO/CN-NPs		Tetracycline IZ (mm)
*Staphylococcus aureus* ATCC 6538	14	125	10	1000	16	62.5	34 ± 0.0
*Listeria monocytogenes* ATCC 19115	14	125	10	1000	16	62.5	37 ± 0.0
Gram-negative bacteria							
*Escherichia coli* ATCC 25922	12	250	8	2000	14	125	30 ± 0.0
*Salmonella enteritidis* DMB 560	12	250	8	2000	14	125	ND

**Table 3:** Anti-leishmanial activity and cytotoxicity of encapsulated *C. verum* EO into CN-NPs.

Samples	IC_50_ ± SD (µg mL^−1^)		CC_50_ ± SD (µg mL^−1^)	SI	
	*L. tropica*	*L. major*	Raw 264.7	*L. tropica*	*L. major*
CN-NPs	NT	NT	>2000	NT	NT
*C. verum* EO	14.11 ± 1.22	17.32 ± 1.22	46.67±0.31	3.30	2.69
*C. verum* EO/CN-NPs opt	10.47± 1.24	15.09± 1.66	1000 ± 2.65	95.5	66.26
AMB	0.34 ± 0.12	0.97 ± 0.08	10.62 ± 0.58	31.23	10.94

CN-NPs: chitosan nanoparticles. IC50: 50% inhibitory concentration; LC50: 50% lethal concentration. NT: not tested. The values presented are the mean of three replicates (*n* = 3) ± standard deviation (SD). SI: selectivity index calculated as the ratio LC50/ IC50. AMB: amphotericin B.

**Table 4:** Characterization of CN-NPs and *C. verum* EO/CN-NPs: average size and surface charge.

Samples	Particle Size (nm ± SD)	Polydispersity	Zeta Potential (mV ± SD)
CN-NPs	210 ± 10.32	0.32	42.57 ± 0.87
*C. verum* EO/CN-NPs opt	480 ± 14.55	0.21	35.64 ± 1.37

## Data Availability

Not applicable.

## Supplementary Materials

The following supporting information can be downloaded at: https://www.mdpi.com/article/10.3390/molecules28155681/s1, Figure S1: Plotted curves of the chosen responses (Encapsulation efficiency and Anti-candida activity MIC) under the experimental space obtained by the axis of the factors ([TPP] (X3), EO/chitosan ratio (X4) and Reaction time (X5)). Figure S2. Individual desirability plot of the encapsulation (Y1) and the anti-candida activity (Y2) responses. Figure S3. Antibacterial activity of C. verum /CS-NPs (1) and CS-NPs (2). Table S1. Plackett-Burman matrix for the chosen responses. Table S2. Coefficient signification of the studied factors for encapsulation efficiency (%) and Anti-candida activity responses. Table S3. Experimental conditions and obtained responses according to Box-Behnken Matrix. Table S4. Statistical signification of the coefficients and the interactions of the studied factors. Table S5. Predicted and observed values of the studied responses for validation runs. Table S6. Studied factors for the two investigated responses: Encapsulation efficiency and anti-candida activity.
