# Supplementary material for: Anti-Candida and Anti-Leishmanial Activities of Encapsulated Cinnamomum verum Essential Oil in Chitosan Nanoparticles
Source: Molecules. 2023 Jul 27;28(15):5681. doi: 10.3390/molecules28155681 (PMC10419485; doi:10.3390/molecules28155681)
Supplement: Supplementary file 1 [file molecules-28-05681-s001.zip › molecules-2388765-supplementary date.pdf]

SUPPLEMENTARY DATA

Anti-Candida and Anti-leishmanial Activities of Encapsulated *Cinnamomum verum* Essential Oil in Chitosan Nanoparticles

Rym Essid[1,*], Ameni Ayed[1], Kais Djebali[2], Houda Saad[3], Mondher Srasra[3], Yasmine Othmani[1], Nadia Fares [1], Selim Jallouli[1], Islem Abid[4], Monerah Rashed Alothman[4], Ferid Limam[1] and Olfa Tabbene[1]

1 Laboratoire des Substances Bioactives, Centre de Biotechnologie de Borj-Cedria, BP 901, Hammam-lif 2050, Tunisia; ayed.amenicbbc@gmail.com (A.A.); yasmine.oth@gmail.com (Y.O.); fares.nadia@yahoo.fr (N.F.); selimjallouli80@gmail.com (S.J.); limam_ferid@yahoo.fr (F.L.); tabb_olfa@yahoo.fr (O.T.)
2 Valorization of Useful MaterialLaboratory (LVMU), National Research Center in Material Sciences (CNRSM) Technopôle Borj Cedria, BP 73, 8027 Soliman – Tunisia/ Tél.:+216 79 325 280 / Fax:+21679 325 314.kais.djebali@gmail.com.
3 Centre National en Recherche en Sciences des Matériaux,"CNRSM" Technopole Borj-Cedria-Route Touristique Soliman, BP-273, Soliman8027,Tunisia;saad_houda@yahoo.com (H.S.); mondher.srasra@gmail.com (M.S.)
4 Department of Botany and Microbiology, College of Science, King Saud University, P.O. 2455, Riyadh11451, Saudi Arabia
* Correspondence: essidrym@hotmail.com; Tel.: +216-71-890-827

Table S1. Plackett-Burman matrix for the chosen responses

No.	Method X_1	Chitosan MW X_2	[TPP] (mg/mL) X_3	*C. verum* EO/chitosan ratio X_4	Reaction time (h) X_5	Encapsulation efficiency Y_1 (%)	Anti-candida activity MIC Y_2 (µg/mL)
1	-1	-1	-1	+1	+1	85.38	2300
2	+1	-1	-1	-1	-1	80.38	2500
3	-1	+1	-1	-1	-1	75.33	2400
4	+1	+1	-1	+1	-1	82.21	2200
5	-1	-1	+1	+1	-1	84.11	1280
6	+1	-1	+1	-1	+1	82.06	580
7	-1	+1	+1	-1	-1	79.26	152.5
8	+1	+1	+1	+1	+1	84.75	307
9	-1	0	0	0	0	90.16	2020
10	+1	0	0	0	0	90.31	2060
11	-1	0	0	0	0	90.74	2010
12	+1	0	0	0	0	90.02	2050
13	-1	0	0	0	0	90.60	2030
14	+1	0	0	0	0	90.60	2070

MW: molecular weigh. [TPP]: TPP concentration.

Table S2. Coefficient signification of the studied factors for encapsulation efficiency (%) and Anti-candida activity responses

Encapsulation efficiency (%)				
Factor name	Regression coefficient	SD[(a)]	t[(b)]	signification
Constant	85.42	0.08	1086.01	<0.01***
Method	0.33	0.08	4.31	1.30*
Chitosan MW	-1.29	0.10	-12.48	<0.01***
[TPP] (mg/mL)	0.86	0.10	8.26	0.10**
C. verum EO/Chitosan ratio	2.42	0.10	23.33	<0.01***
Reaction time (h)	0.19	0.10	1.88	13.30
Anti-candida activity MIC (µg/mL)				
Factor name	Regression coefficient	SD	t	signification
Constant	1711.39	2.67	640.34	<0.01***
Method	-30.39	2.67	-11.37	<0.01***
Chitosan MW	-200.06	3.53	-56.58	<0.01***
[TPP] (mg/mL)	-885.06	3.53	-250.33	<0.01***
C. verum EO/Chitosan ratio	56.81	3.53	16.06	<0.01***
Reaction time (h)	-68.18	3.53	-19.28	<0.01***

C. verum EO: Cinnamon verum essential oil

Table S3. Experimental conditions and obtained responses according to Box-Behnken Matrix

No. exp	[TPP] (mg/mL)	*C. verum* EO/chitosan ratio	Reaction time (h)	Encapsulation efficiency (%)	Anti-candida activity MIC (µg/mL)
1	-1	-1	0	82.56	1200
2	+1	-1	0	80.23	600
3	-1	+1	0	83.35	240
4	+1	+1	0	92.58	125
5	-1	0	-1	74.19	2700
6	+1	0	-1	62.90	4000
7	-1	0	+1	79.20	1200
8	+1	0	+1	81.44	1000
9	0	-1	-1	60.33	1500
10	0	+1	-1	73.25	1100
11	0	-1	+1	70.33	3000
12	0	-1	+1	85.61	500
13	0	0	0	80.31	2400
14	0	0	0	80.25	2420
15	0	0	0	81.12	2440
16	0	0	0	81.54	2350
17	0	0	0	80.38	2390
18	0	0	0	80.14	2450
19	0	0	0	80.46	2480
20	0	0	0	81.81	2430

Table S4. Statistical signification of the coefficients and the interactions of the studied factors

Factors	Encapsulation				Anti-candida activity MIC			
	Value (%)	SD	t	signification	Value (µg/mL)	SD	t	Signification
Mean/Interc.	80.98	0.33	245.49	<0.01***	2414.00	18.05	133.69	<0.01***
[TPP] (L) a_3	-0.26	0.26	-1.03	36.10	48.12	14.27	3.37	2.801*
[TPP] (Q) a_{33}	2.87	0.35	7.99	0.13	-586.37	19.67	-29.80	<0.01***
C. verum EO/chitosan ratio (L) a_4	5.16	0.26	19.81	<0.01***	-541.87	14.27	-37.96	<0.01***
C. verum EO/chitosan ratio (Q) a_{44}	0.82	0.35	2.28	8.42	-1286.38	19.67	-65.38	<0.01***
Reaction time (L) a_5	5.73	0.26	22.00	<0.01***	-450.00	14.27	-31.52	<0.01***
Reaction time (Q) a_{55}	-9.42	0.36	-26.22	<0.01***	397.38	19.67	20.19	<0.01***
a_{34}	2.89	0.37	7.83	0.14**	121.25	20.18	6.00	0.387**
a_{35}	3.38	0.37	9.17	0.08**	-375.00	20.18	-18.57	<0.01***
a_{45}	0.59	0.37	1.60	18.49	-525.00	20.18	-26.00	<0.01***

(***, ** and*): level of significance of (<0.01%, <1% and<5% respectively); ***L***, ***Q***: linear and quadratic terms.

Table S5. Predicted and observed values of the studied responses for validation runs

Optimal conditions		
Factor name	Level	Value
[Tpp] (mg/mL)	+1	1
C. verum EO/chitison ratio	+1	2
Reaction time (h)	+0.5	2.5
Chosen responses		
	Encapsulation efficiency (%)	Anti-candida activity MIC (µg/mL)
Predicted	95	115
Observed	93 ± 3	120 ± 9.83

Table S6. Studied factors for the two investigated responses: Encapsulation efficiency and anti-candida activity

Factors name	Unit	Code	Coded levels		
			-1	0	+1
Method	-	X_1	A	-	B
Chitosan MW	-	X_2	Low	Medium	High
[TPP]	mg/mL	X_3	0.05	0.525	1
C. verum EO/chitosan ratio	-	X_4	0.25	1.125	2
Reaction time (h)	h	X_5	1	2	3

MW: molecular weigh

C. verum EO: *Cinnamon verum* essential oil

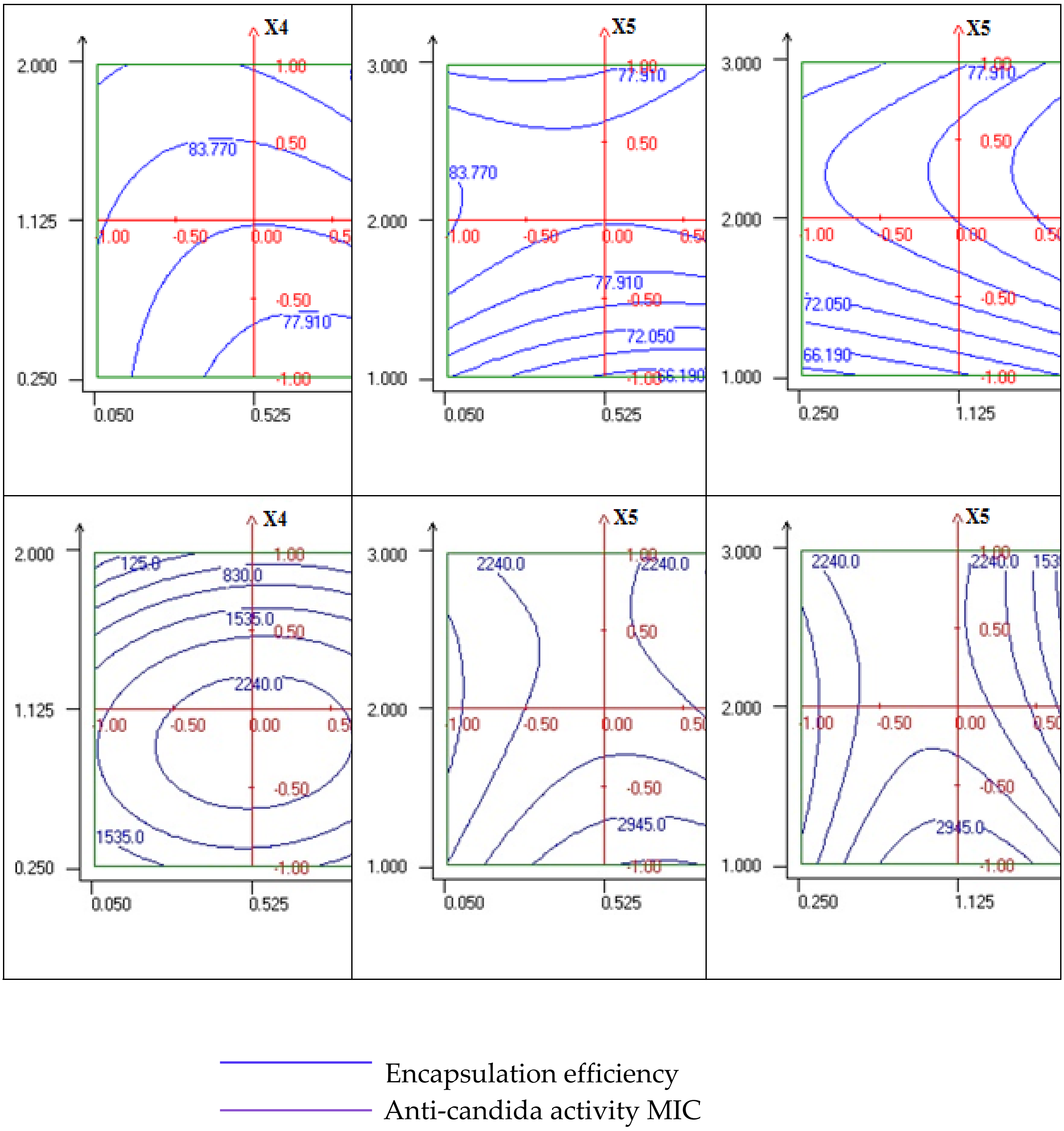

Figure S1: Plotted curves of the chosen responses (Encapsulation efficiency and Anti-candida activity MIC) under the experimental space obtained by the axis of the factors ([TPP] (**X**$_3$), EO/chitosan ratio (**X**$_4$) and Reaction time (**X**$_5$))

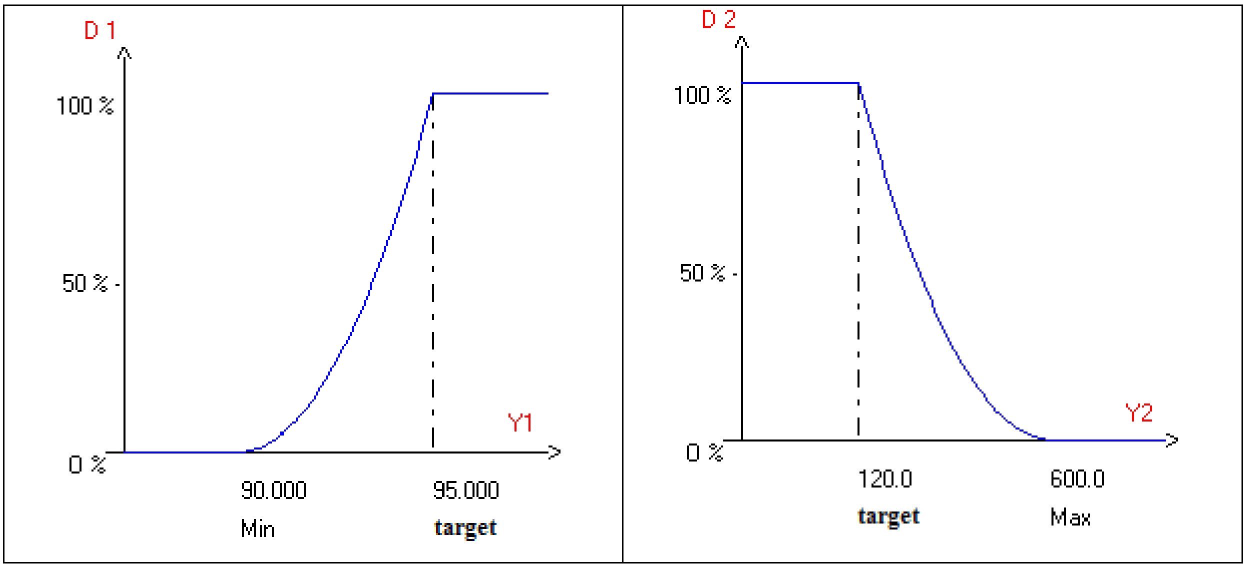

Figure S2. Individual desirability plot of the encapsulation (**Y**$_1$) and the anti-candida activity (**Y**$_2$) responses

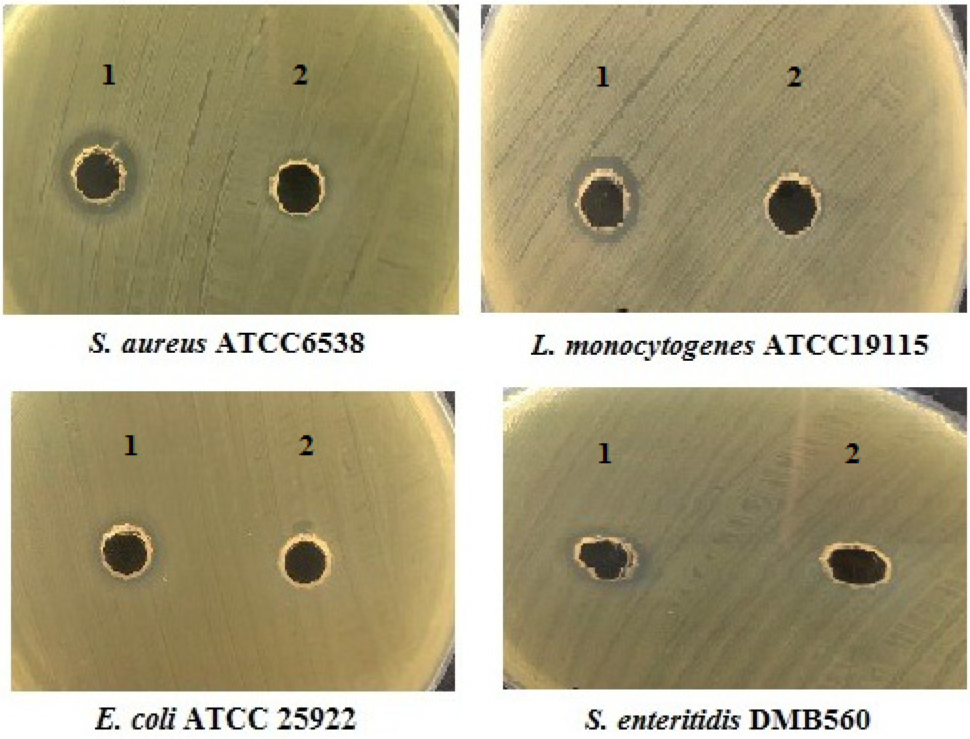

Figure S3. Antibacterial activity of *C. verum* EO/CN-NPs (1) and CN-NPs (2)
